# Why should early-career scientists publish in society journals

**DOI:** 10.1128/mbio.01994-23

**Published:** 2023-12-13

**Authors:** Stephen K. Dolan, Lori D. Banks, Wenqi Yu

**Affiliations:** 1 Department of Genetics and Biochemistry, Eukaryotic Pathogens Innovation Center, Clemson University, Clemson, South Carolina, USA; 2 Department of Biology, Prairie View A&M University, Prairie View, Texas, USA; 3 Department of Molecular Bioscience, University of South Florida, Tampa, Florida, USA; Johns Hopkins Bloomberg School of Public Health, Baltimore, Maryland, USA

**Keywords:** society journals, early career scientists, impact

## Abstract

In this editorial, written by early-career scientists, we advocate for the invaluable role of society journals in our scientific community. By choosing to support these journals as authors, peer reviewers, and as editors, we can reinforce our academic growth and benefit from their re-investment back into the scientific ecosystem. Considering the numerous clear merits of this system for future generations of microbiologists and more broadly, society, we argue that early-career researchers should publish our high-quality research in society journals to shape the future of science and scientific publishing landscape.

## EDITORIAL

Society journals are deeply rooted in our scientific community and a vital part of it. They serve as a venue for the publication of high-quality articles while promoting the mission-driven activities of their respective societies (1). While these journals play an important role in facilitating scientific communication among scientists, the activities of the associated scientific societies go beyond this; they invest in our growth.

Examples of this include the funding of conferences which enables us to showcase our best work, awards for young scientists, the provision of generous travel grants for scientists with limited financial resources, and training programs to improve the quality of scientific research, science communication, and peer review. Their support extends to scientists at varied career stages and across the world, providing all with the ability to directly impact the scientific community. These initiatives are particularly impactful for early-career scientists; for example, junior editor programs provide opportunities to acquire lifelong skills for reviewing research while also honing writing and editing skills. Scientific societies and their journals also have the power to address important issues that directly impact early-career researchers worldwide. For instance, they can be intentional in recruiting and maintaining a diverse group of reviewers and staff to ensure that a wide range of perspectives enrich our joint scientific endeavor.

Society journals have rigorous review process which is led by field-specific expert scientists as editors and reviewers. The editors and reviewers are the scientific publishing gatekeepers, responsible for evaluating the quality, validity, and significance of submissions. Society journals emphasize the quality of research by having expert scientists as editors and reviewers. Rigorous experts reviewing process with high standards ensure that only well-designed and sound work is published in society journals. In comparison, for-profit journals may emphasize novelty and “hot” topics; predatory publishers may seek sheer volume to collect article processing charges (APCs). Another compelling feature of society journals is their diverse range of article types. Research articles with significant and broad impact as well as articles reporting negative data (e.g., high-quality data that do not support the initial hypothesis or studies which have failed to find statistically significant results) can all find a home in dedicated society journals. All these features of society journals greatly serve scientific transparency and communication and advancement of science.

For society journals to finance their operations and make all the above possible, they rely on the authors who publish their work in these journals to pay APCs. What sets them apart from most other publishers is the direct investment of any surplus into supporting their strategic missions, which for the American Society for Microbiology (ASM) means to “promote and advance the microbial sciences.” It is a virtuous cycle where the community benefits from the research that is published. In contrast, for-profit commercial publishers use subscription fees or APCs to cover publishing costs, and the remainder is used to generate substantial profits for shareholders. The academic publishing industry has one of the highest profit margins known, often exceeding 40% (2, 3). These funds are, in effect, leaving the scientific ecosystem. Considering that academic research is a heavily publicly subsidized product (with articles peer reviewed by the scientific community free of charge and APCs largely paid by taxpayers), these profits should be alarming.

If publishing in society journals has all the positives described above, then why is not most of our microbiology research published in society journals? It appears that aggrandized journal impact factors (JIFs), widely advertised “invited guest editor special issues,” and, in some cases, rapid peer review attract researchers to submit to for-profit mega journals over society journal counterparts. In addition, the expansion of new subsidiary and partner journals, which leverage the prestigious titles of flagship journals or publication houses, directly compete with society journals (4).

Does JIF correlate with research quality? Echoing many excellent reviews on this topic, journal-level metrics (particularly the JIF) do not reflect the impact or influence of individual research articles (5). The complete disconnect between JIFs and article quality led to a collective decision by ASM Journals (and other non-profit journals) to eliminate any mention of the JIF on the journals’ website (6). As mentioned earlier, society journals hold a high standard of scientific rigor by having discipline-specific experts as editors and reviewers. The quality of the articles published in society journals is consistently high, a metric which cannot be reflected by a simple JIF.

**Fig 1:**
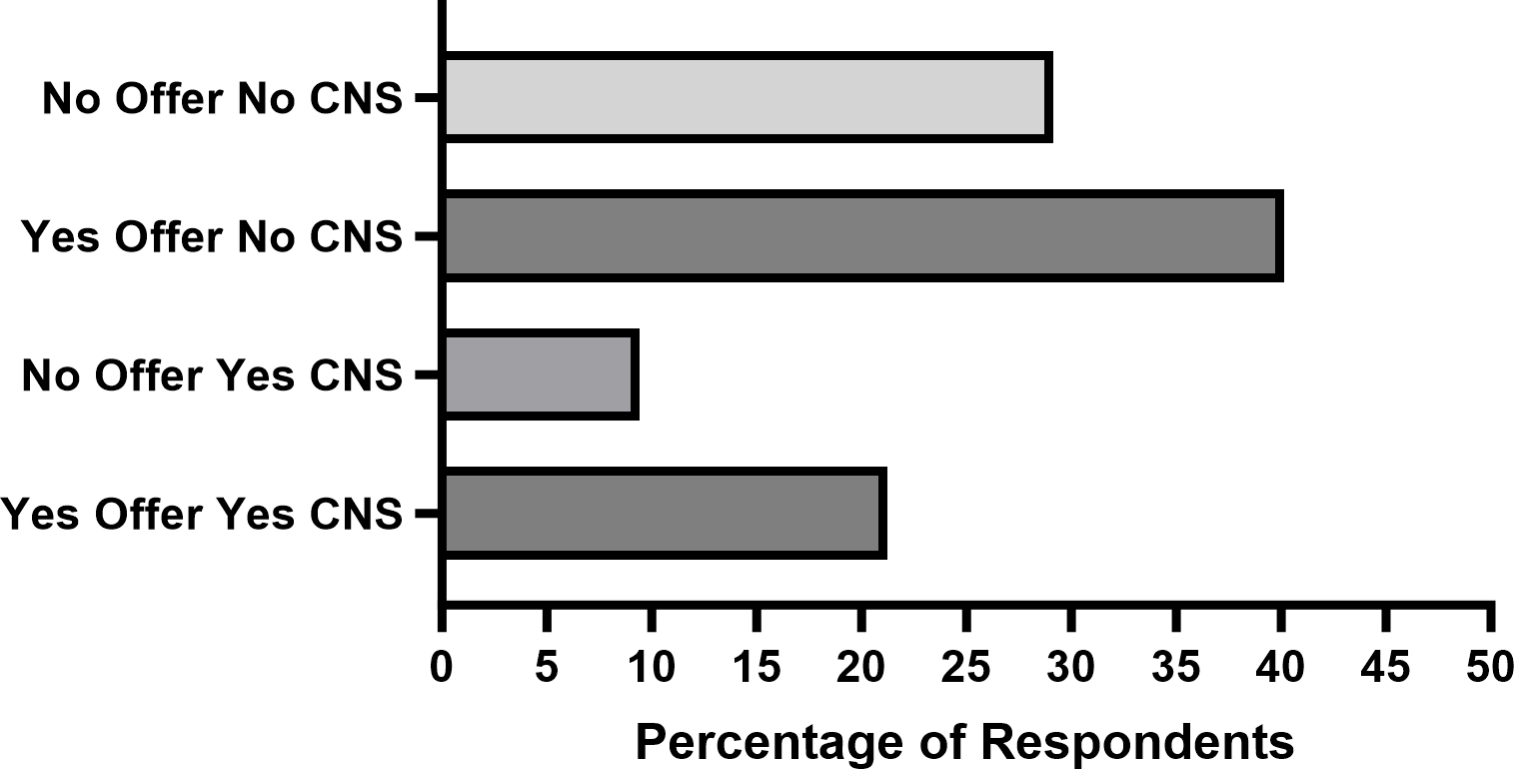
Self-reported faculty job offers as a function of CNS publications. Self-reported job offers and publication statistics from 127 respondents from the New PI Slack group. CNS publications are those occurring in the flagship journal of *Cell*, *Nature*, or *Science* or a subsidiary journal of those listed above.

Do publications in for-profit subsidiary journals result in more status, prestige, or career opportunities for early-career researchers when compared to publishing in leading society or non-profit journals? To explore this question, we gathered survey data from volunteers in the “New PI” Slack channel and analyzed the responses of 127 individuals who reported to have been applying for faculty positions in the 2022–2023 hiring season. The majority (40%) of the respondents got a job offer without having a paper in the journals *Cell*, *Nature*, and *Science* (CNS) or their subsidiary journals (Fig. 1). In comparison, about 10% of the respondents did not get a job offer while having a CNS or CNS subsidiary journal paper (Fig. 1). We note that the for-profit journals *Cell* and *Nature* and their subsidiaries are amalgamated with the non-profit publisher *Science* in this data set. There are many other metrics that were not covered in this survey, such as numbers of job interviews, demographics, specific details of the job offer, personal situations, etc. Nevertheless, the data are largely consistent with the results from a previous survey showing that although a first authorship on a flagship CNS paper can result in a higher percentage of offers per job application submitted, having a CNS paper is far from a necessity to secure a faculty job offer (7). Importantly, a first authorship in a for-profit subsidiary journal does not have a quantifiable impact on a faculty job offer success rates.

In conclusion, scientific societies and their associated journals are an asset to our community and are excellent homes for your best research work. These journals are known for rigorous peer view, ethical, and transparent practices which enhance the credibility of the published work and contribute to the overall integrity of science. The profits generated from publishing with these societies are re-invested into the scientific community, benefiting early-career scientists. It is encouraging to see organizations like International Society for Microbial Ecology moving their flagship journals *The ISME Journal* and *ISME Communications* from paywalled, for-profit publication to the non-profit publisher Oxford University Press beginning in 2024 (8). Early-career scientists may feel they have limited influence over longstanding issues in the academic publishing model, but it is essential to ask ourselves how our journal choices today will shape the future scientific publishing landscape.
